# Prevalence and risk factors of general and abdominal obesity and hypertension in rural and urban residents in Bangladesh: a cross-sectional study

**DOI:** 10.1186/s12889-022-14087-8

**Published:** 2022-09-08

**Authors:** Nurshad Ali, Nayan Chandra Mohanto, Shaikh Mirja Nurunnabi, Tangigul Haque, Farjana Islam

**Affiliations:** Department of Biochemistry and Molecular Biology, Shahjalal University of Science and Technology, Sylhet, 3114 Bangladesh

**Keywords:** General obesity, Abdominal obesity, Hypertension, Rural and urban, Risk factors, Adults, Bangladesh

## Abstract

**Background:**

Obesity and hypertension are global health concerns. Both are linked with increased risks of all-cause and cardiovascular mortality. Several early studies reported the prevalence of obesity and hypertension in Bangladeshi adults, but the associated factors in this country population are not clear yet. We aimed to estimate the prevalence and related risk factors of general and abdominal obesity and hypertension in rural and urban adults in Bangladesh.

**Methods:**

In this cross-sectional study, data (*n* = 1410) was collected on rural (*n* = 626) and urban (*n* = 784) adults from eight divisional regions of Bangladesh. Both anthropometric and socio-demographic measurements were recorded in a standardized questionnaire form. General and abdominal obesity were defined based on WHO proposed cut-off values and hypertension was defined by SBP ≥ 140 mmHg and/or, DBP ≥ 90 mmHg and/or, intake of anti-hypertensive drugs at the time of data collection. Multivariable logistic regression analyses were performed to assess the relationship of general and abdominal obesity and hypertension with various factors.

**Results:**

The overall prevalence of general obesity, abdominal obesity and hypertension was 18.2, 41.9 and 30.9%, respectively. The women had a higher prevalence of general obesity (25.2%), abdominal obesity (56.1%) and hypertension (32.3%) compared to the men (12.2, 29.0, and 29.7%, respectively). The prevalence of both general and abdominal obesity was higher in urban participants (21.7 and 46.6%, respectively) than in the rural participants (13.8 and 35.1%, respectively), whereas, the rural participants had a higher prevalence of hypertension (35.1%) compared to the urban participants (27.5%). In geographical region comparison, the prevalence of general and abdominal obesity and hypertension were higher in participants enrolled from Dhaka (30.8%), Khulna (63.6%) and Mymensingh (43.5%) regions, respectively compared to other regions. In regression analysis, increased age, place of residence and less physical activity were positively associated with the increased risk of both types of obesity and hypertension. The analysis also showed a significant positive association between high BMI and an increased risk of hypertension.

**Conclusion:**

This study shows a high prevalence of obesity and hypertension in rural and urban adults. Increased age, inadequate physical activity and place of residence were significant determinants of general and abdominal obesity and hypertension. A comprehensive intervention program focusing on modifiable risk factors such as lifestyles and food habits is needed to increase awareness and prevent the burden of obesity and hypertension in the Bangladeshi population.

## Background

The prevalence of both obesity and hypertension is increasing speedily in the world and has been considered a public health concern. Approximately, 603 million adults were found to be obese globally in 2015, and this number has risen gradually since 1980 [1]. Obesity is generally measured using body mass index (BMI) [2, 3], which has been proven to increase the risk of hypertension, coronary heart disease, stroke, diabetes and other non-communicable diseases [4]. However, BMI alone cannot provides complete information on body fat distribution, which is associated with metabolic risk [5]. Moreover, BMI often fails to assess the cardiometabolic risk in adults with an excess of adiposity [5]. In this case, abdominal obesity which is measured based on waist circumference (WC) or waist-to-hip ratio can provide useful information on visceral fat accumulation in the body [6]. Abdominal obesity is associated with an increased risk of type 2 diabetes, cardiovascular disease, metabolic syndrome and all-cause mortality [7, 8].

On the other hand, hypertension is a major cause of morbidity and mortality. A large-scale study used data from 1990 to 2019 on people aged 30–79 years and reported the global prevalence of hypertension at 34% in men and 32% in women [9]. Hypertension is a significant risk factor for disability and death worldwide, affecting more than 1 billion people and causing approximately 9.4 million deaths every year [10, 11]. In contrast to developed countries, the prevalence of hypertension is also increasing in developing countries with no improvement in awareness or control measures [12]. In Asia, especially the South Asian countries are facing a burden of hypertension and associated diseases [13, 14].

Studies showed that obesity and hypertension are often occurred together [15] and increased the risk of cardiovascular mortality [16, 17]. It has been reported that both demographic and socioeconomic transitions have contributed to the burden of obesity and hypertension in developing countries [18, 19] and the epidemiologic transition from infectious diseases to non-infectious diseases [20, 21]. Bangladesh is a developing country in South Asia, with the rapid urbanization and industrialization in recent years, the prevalence of obesity and hypertension has increased remarkably in this country. Several early studies reported the prevalence of obesity and hypertension in Bangladeshi adults [22–27]; however, a number of them were age and area specific and focused on either obesity or hypertension. Moreover, the factors associated with the risk of obesity and hypertension in Bangladeshi adult populations are not clear yet. Therefore, this study aims were to measure the prevalence of both general and abdominal obesity and hypertension and identify its associated risk factors in rural and urban adults from all divisional regions of Bangladesh.

## Methods

### Study subjects and study areas

This study was a cross-sectional design conducted between September 2017 and April 2018. Bangladesh has 8 administrative or divisional regions. Data was collected on 1410 participants (761 males and 649 females) from rural (*n* = 626) and urban (*n* = 784) communities of all these regions. The subjects (aged ≥18 years) who were consented to participate, included in the present study. We followed a systematic sampling procedure and we selected every 10th household for participation. Then we considered only one family member from each house for participation. We also examined an equal probability during household selection so that the selected households can provide a statistically reliable estimate of major anthropometric and health-related variables. The inclusion criteria were both genders, age ≥ 18 years, willingness to participate and free from severe chronic illness. We also set some exclusion criteria, for example, we did not collect data on participants who were pregnant or nursing mothers, and individuals who had hepatic disease, cardiac disease, renal disease and malignant disease. The participants with missing socio-demographic information were also excluded from the study. All subjects were informed about the study objectives and written consent was obtained from them before inclusion in the study. This study protocol was reviewed and approved by the Internal Ethics Review Committee existed at the Department of Biochemistry and Molecular Biology of the university.

### Data collection

The data were collected on demographic, socioeconomic and lifestyle factors using a pre-structured questionnaire. The questionnaire was administered by trained interviewers at participants’ homes. Anthropometric data like weight, height, waist circumference (WC) and hip circumference (HC) were measured following standard procedure described elsewhere [28–34]. We asked all the participants to avoid tea, coffee, beverages, eating, smoking and heavy physical work at least 20 min before blood pressure (BP) measurement. The participants were also allowed for 10 min rest before measuring BP three times at 5 min intervals on the left arm in a comfortable sitting position using a digital BP machine (Omron M10, Tokyo, Japan). The first measurement was discarded and then the mean value of the second and third measurements was counted for systolic blood pressure (SBP) and diastolic blood pressure (DBP). At the end of height, weight and BP measurement, we informed all the participants about their body mass index (BMI, weight in kg divided by height in meter squared) and BP status with health messages in Bengali, the local language. The health messages included information on risk factors of obesity and hypertension for example less physical activity, uncontrolled blood pressure, fatty diet, lifestyle and smoking.

### Definitions

Body mass index (BMI) (kg/m^2^) was categorized as underweight (BMI < 18.5), normal (BMI 18.5–23.5), overweight (BMI 23.5–27.5) and obese (BMI > 27.5) according to WHO guideline for Asian population [35, 36]. Abdominal obesity was defined as a WC ≥ 80 cm for females and ≥ 90 cm for males [35, 37]. Hypertension was defined by systolic blood pressure (SBP) ≥ 140 mmHg and/or, diastolic blood pressure (DBP) ≥ 90 mmHg and/or, intake of anti-hypertensive drugs at the time of data collection [38, 39]. Prehypertension was defined as SBP 120–139 mmHg; and/or DBP 80–89 mmHg [38, 39]. The level of education was graded as illiterate who were unable to write and read, primary or elementary, secondary, higher secondary and above. Participant’s socioeconomic status was classified based on their household assets, properties and per month income (high: > 20,000 Bangladeshi Taka, BDT, medium: 10000–20,000 BDT and low: < 10,000 BDT) [1 USD = 85 BDT). Physical activity was graded as low (comfortable housework and official work), medium (walking, swimming and household stuff cleaning) and adequate/high (jogging, carrying, lifting, and/or sports). Smoking status was defined as a never smoker and present smoker.

### Statistical analysis

Descriptive data were presented as mean and SD for the continuous variables and frequency and percentages for the categorical variables. A chi-square test was applied to assess the proportional differences in obesity and hypertension status in the categorical variables. Independent sample t-test and one-way ANOVA were used to determine the differences between anthropometric and socio-demographic variables. Bivariate and multivariable logistic regression models were conducted to identify the significant risk factors for obesity and hypertension. In the multivariable regression analysis, we have adjusted for the covariates age, sex, place of living regions, BMI, education, socioeconomic status, physical activity, family history of obesity and hypertension, intake of raw salt, and smoking. All statistical analyzes were performed using IBM SPSS Statistics version 23. The significance level was set at *p* < 0.05.

## Results

### Characteristics of the study participants

A summary of the demographic and socioeconomic characteristics of the study subjects is presented in Table 1. Out of 1410 participants, 53.9% were male and 46.1% were female. The mean age, BMI and WC of the participants were 39.0 ± 14.2 years, 23.8 ± 4.2 kg/m^2^, and 77.1 ± 24.1 cm, respectively. The mean SBP and DBP were 125.5 ± 17.8 mmHg and 79.3 ± 27.1 mmHg, respectively. Overall, an important portion of the participants had no formal education (15.7%). About 29, 56 and 15% of the participants were in the low, medium and high socioeconomic status groups, respectively. Only 10% of participants were used to high or adequate physical activity, whereas, 42.6% of participants were used to low physical activity. About 40% of the participants had a history of hypertension in their family and 47.2% of participants had no proper knowledge about hypertension. The knowledge about hypertension was higher in males (55.3%) than in women (49.7%) and it was higher among urban residents (61.4%) than the rural residents (37.9%). In our survey, about 20% of participants were smokers.

**Table 1:** Descriptive characteristics for socio-demographic and anthropometric data by geographic region

Measure	Total	Barisal	Chittagong	Dhaka	Khulna	Mymensingh	Rajshahi	Rangpur	Sylhet	*P*-value
*N*	1410	145	182	224	152	169	210	158	170	–
Gender (m/f)	761/649	69/76	88/94	107/117	58/94	112/57	127/83	63/95	114/56	–
Age (years)	39.0 ± 14.2	39.5 ± 15.1	38.7 ± 11.9	37.1 ± 13.3	41.9 ± 15.9	40.4 ± 13.4	43.1 ± 15.0	36.2 ± 13.1	36.0 ± 15.2	0.000
BMI (kg/m^2^)	23.8 ± 4.2	24.5 ± 4.3	23.4 ± 3.8	25.9 ± 3.5	23.8 ± 4.5	23.7 ± 4.2	23.4 ± 4.0	21.5 ± 4.4	23.0 ± 4.2	0.000
WC (cm)	77.1 ± 24.1	83.1 ± 10.7	56.4 ± 31.7	83.1 ± 16.8	88.2 ± 11.0	83.3 ± 15.7	80.6 ± 15.4	56.1 ± 39.2	82.8 ± 15.0	0.000
HC (cm)	159.3 ± 8.9	158.4 ± 9.3	163.4 ± 6.9	158.5 ± 8.0	157.2 ± 9.4	159.4 ± 10.5	159.2 ± 8.0	155.8 ± 9.5	161.3 ± 8.6	0.000
WHR	0.89 ± 0.31	0.94 ± 0.04	0.69 ± 0.37	0.90 ± 0.08	0.93 ± 0.06	1.00 ± 0.75	0.91 ± 0.09	0.92 ± 0.07	0.91 ± 0.11	0.000
SBP (mmHg)	125.5 ± 17.8	125.8 ± 20.5	117.7 ± 13.1	120.1 ± 15.9	128.3 ± 19.4	130.7 ± 14.5	129.3 ± 17.9	131.1 ± 20.3	126.2 ± 17.4	0.000
DBP (mmHg)	79.3 ± 27.1	78.8 ± 12.4	77.2 ± 8.2	76.4 ± 11.9	80.0 ± 11.4	78.5 ± 17.3	79.7 ± 10.5	83.6 ± 12.5	76.9 ± 11.2	0.001
PP (mmHg)	69.8 ± 30.2	80.6 ± 11.5	78.2 ± 10.2	79.6 ± 15.9	81.9 ± 13.3	78.8 ± 18.3	77.3 ± 17.2	86.2 ± 12.8	78.4 ± 11.6	0.000
Place of residence (%)										0.000
Rural	44.4	47.9	45.8	33.9	52.3	39.5	72.9	77.5	45.3	
Urban	55.6	52.1	54.2	66.1	47.7	60.5	27.1	22.5	54.7	
Occupation (%)										0.000
Farmer	6.1	7.9	2.9	5.6	1.0	2.0	9.1	1.1	1.1	
Housewives	29.5	31.7	44.9	39.8	26.5	34.0	24.4	17.6	5.7	
Business	10.6	15.9	6.6	18.9	11.8	1.0	7.3	18.7	2.9	
Job	37.0	27.0	30.9	28.6	11.8	35.0	39.0	49.5	79.0	
Others	10.7	17.5	14.7	7.1	49.0	10.0	20.1	12.2	11.4	
Education (%)										0.000
Illiterate	15.7	15.9	9.0	18.9	15.4	31.6	21.2	8.6	2.0	
Primary	18.8	3.2	27.6	26.0	13.5	21.4	21.2	15.1	7.9	
Secondary	16.1	17.5	16.4	24.0	8.7	11.2	17.0	18.3	11.8	
Higher Secondary	13.7	34.9	11.9	7.7	13.5	10.2	15.2	20.4	12.8	
Above Higher Sec.	35.6	28.6	35.1	23.5	49.0	25.5	25.5	37.6	64.5	
Socioeconomic status (%)										0.000
Low	28.8	19.0	22.6	25.0	37.9	34.7	52.7	25.0	4.2	
Medium	56.0	74.6	58.3	61.4	45.5	44.9	34.2	66.7	65.0	
High	15.1	6.3	19.1	13.6	16.7	20.4	13.0	8.3	28.8	
Family history of hypertension (%)										0.000
Yes	40.0	43.3	52.6	20.6	33.3	38.5	42.7	51.6	52.4	
No	59.5	56.7	47.4	79.4	66.7	61.5	57.3	48.4	49.0	
Knowledge about hypertension										0.000
Yes	52.8	36.7	89.7	86.6	34.3	28.2	13.3	44.4	24.6	
No	47.2	63.3	10.3	13.4	65.7	71.8	86.7	55.6	75.4	
Physical activity (%)										0.000
Low	42.6	3.3	2.5	52.2	94.0	95.0	96.5	65	10.0	
Moderate	47.4	36.7	87.9	47.8	6.0	5.0	3.5	35	90.0	
Adequate	10.0	60.3	10.3	0.0	0.0	0.0	0.0	0.0	0.0	
Smoking status (%)										0.000
Yes	20.4	10.4	10.6	22.8	25.7	30.7	29.6	28.9	15.8	
No	79.6	89.6	89.4	77.2	74.3	69.3	70.4	71.1	84.2	

Data are presented as mean ± SD for the continuous variables and percentages for the categorical variables. *P*-values are obtained from one way ANOVA for continuous variables and from the chi-square test for categorical variables

### Body mass index and waist circumference data

**Fig. 1:**
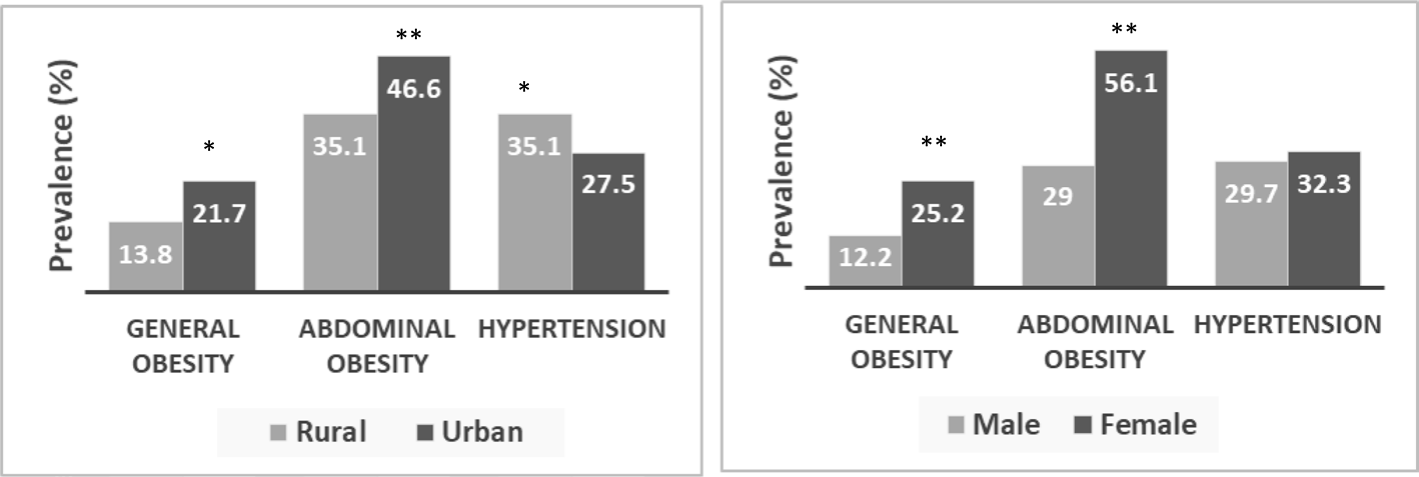
Prevalence of general obesity, abdominal obesity and hypertension in adults by region and gender. *P < 0.01, **P < 0.001 when the prevalence is compared between the region and gender groups. *P*-values are obtained from the chi-square test

Table 2, Table 3 and Fig. 1 summarize the BMI and WC data by gender and region. The prevalence of general obesity was 18.2% and abdominal obesity was 41.9%. About 9% of the participants were underweight and 39% were overweight. Both general and abdominal obesity prevalence was higher in females (25.2 and 56.1%, respectively) than the males (12.2 and 29%, respectively). Similarly, both types of obesity were higher in urban residents (21.7 and 46.6%, respectively) than in rural residents (13.8 and 35.1%, respectively) (*p* < 0.01 and *p* < 0.001, respectively). In geographical region comparison, general and abdominal obesity prevalence was higher among participants from Dhaka (30.8%) and Khulna (63.6%) regions, respectively. The lowest percentage of general and abdominal obesity was found in Rangpur and Chittagong regions (9, 18.3%, respectively).

**Table 2:** Characteristics of the study subjects by BMI, WC and blood pressure in rural and urban adults of different regions

Divisions	Living area	Body mass index				Waist circumference		Blood pressure		
		Underweight (%)	Normal (%)	Overweight (%)	Obesity (%)	Normal (%)	Obesity (%)	Normal (%)	Pre-hypertensive (%)	Hypertensive (%)
Barisal	Rural	6.5	26.1	41.3	26.1	60.9	39.1	39.1	26.1	34.8
	Urban	6.0	36.0	36.0	22.0	48.0	52.0	44.0	30.0	26.0
	Total	6.3	31.3	38.5	23.9	54.2	45.8	41.7	28.1	30.2
Chittagong	Rural	0.0	27.7	44.6	27.7	94.1	5.9	7.7	43.1	49.2
	Urban	2.6	64.9	29.9	2.6	76.0	24.0	55.8	20.8	23.4
	Total	1.4	47.9	36.6	14.1	81.7	18.3	33.8	31.0	35.2
Dhaka	Rural	0.0	22.4	50.0	27.6	55.5	44.5	60.0	20.0	20.0
	Urban	1.4	18.2	47.9	32.4	52.1	47.9	42.0	39.3	18.7
	Total	0.9	19.6	47.7	30.8	53.8	46.2	42.4	38.8	18.8
Khulna	Rural	12.5	41.1	28.6	17.9	35.7	64.3	28.6	33.9	37.5
	Urban	7.8	33.3	37.3	21.6	37.3	62.7	27.5	35.3	37.3
	Total	10.3	37.4	32.7	19.6	36.4	63.6	28.0	34.6	37.4
Mymensingh	Rural	10.2	44.9	28.6	16.3	61.7	38.3	20.4	32.7	46.9
	Urban	8.0	33.3	38.7	20.0	42.7	57.3	16.0	42.7	41.3
	Total	8.9	37.9	34.7	18.5	50.0	50.0	17.7	38.7	43.5
Rajshahi	Rural	13.7	39.5	41.9	4.8	75.0	25.0	25.0	48.4	26.6
	Urban	4.3	26.1	37.0	32.6	48.8	51.2	23.9	37.0	39.1
	Total	11.2	35.9	40.6	12.4	68.3	31.7	24.7	45.3	30.0
Rangpur	Rural	29.1	43.0	20.9	7.0	64.7	35.3	23.3	37.2	39.5
	Urban	4.0	40.0	40.0	16.0	60.0	40.0	16.0	36.0	48.0
	Total	23.4	42.3	25.2	9.0	63.2	36.8	21.6	36.9	41.4
Sylhet	Rural	24.2	35.5	32.2	8.1	62.3	37.7	33.9	45.2	21.0
	Urban	9.3	25.3	52.0	13.3	60.3	39.7	33.3	42.7	24.0
	Total	16.1	29.9	43.1	10.9	61.2	38.8	31.2	37.9	30.9

Data are presented as percentages from the chi-square test. (BMI) (kg/m^2^) was categorized as underweight (BMI < 18.5), normal (BMI 18.5–23.5), overweight (BMI 23.5–27.5) and obese (BMI > 27.5) [35, 36]. Abdominal obesity was defined as a WC ≥ 80 cm for females and ≥ 90 cm for males [35, 37]. Hypertension was defined as SBP ≥ 140 mmHg and/or, DBP ≥ 90 mmHg and/or, intake of anti-hypertensive drugs [38, 39]. Prehypertension was defined as SBP 120–139 mmHg; and/or DBP 80–89 mmHg [38, 39]

**Table 3:** Characteristics of the study subjects by BMI, WC and blood pressure data

Living area	Gender	n	Body mass index				Waist circumference		Blood pressure		
			Underweight (%)	Normal (%)	Overweight (%)	Obesity (%)	Normal	Obesity	Normal (%)	Pre-hypertensive (%)	Hypertensive (%)
Rural	Male	340	11.2	42.5	36.6	9.7	74.9	25.1	22.0	43.3	34.7
	Female	286	18.7	31.1	31.6	18.7	54.1	45.9	28.9	35.6	35.6
	Total	626	14.6	37.3	34.3	13.8	64.9	35.1	25.2	39.8	35.1
Urban	Male	421	6.0	34.3	45.5	14.2	68.3	31.7	31.6	42.8	25.6
	Female	363	2.4	28.0	39.2	30.4	36.4	63.6	41.3	29.0	29.7
	Total	784	4.4	31.4	42.6	21.7	53.4	46.6	36.1	36.4	27.5
Overall	Male	761	8.3	38.0	41.5	12.2	71.0	29.0	27.3	43.0	29.7
	Female	649	9.6	29.4	35.8	25.2	43.9	56.1	35.8	31.9	32.3
	Total	1410	8.9	34.0	38.9	18.2	58.1	41.9	31.2	37.9	30.9

Data are presented as percentages obtained from the chi-square test. (BMI) (kg/m^2^) was categorized as underweight (BMI < 18.5), normal (BMI 18.5–23.5), overweight (BMI 23.5–27.5) and obese (BMI > 27.5) [35, 36]. Abdominal obesity was defined as a WC ≥ 80 cm for females and ≥ 90 cm for males [35, 37]. Hypertension was defined as SBP ≥ 140 mmHg and/or, DBP ≥ 90 mmHg and/or, intake of anti-hypertensive drugs [38, 39]. Prehypertension was defined as SBP 120–139 mmHg; and/or DBP 80–89 mmHg [38, 39]

### Blood pressure data

**Fig. 2:**
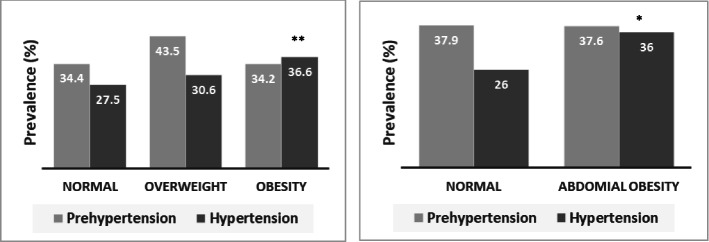
Prevalence of hypertension in the BMI and WC groups *P < 0.05 when hypertension prevalence is compared between the WC groups, **P < 0.01 when hypertension prevalence is compared within BMI groups. *P*-values are obtained from the chi-square test

The blood pressure data for all participants are summarized in Table 2, Table 3 and Fig. 1 by gender, place of residence and geographic region. The overall prevalence of prehypertension and hypertension was 37.9 and 30.9%, respectively. Females were more hypertensive (32.3%) than males (29.7). However, the prehypertension rate was higher in males (43%) than the females (31.9%). Both prehypertension and hypertension prevalence were higher among participants living in rural areas (39.8 and 35.1%, respectively) than those living in urban areas (36.4 and 27.5%, respectively). The prevalence of obesity and hypertension varied in the geographic regions. The lowest and highest prevalence of hypertension was observed in Dhaka (18.8%) and Mymensingh (43.5%) region, respectively. In the BMI groups, the prevalence of prehypertension and hypertension was significantly higher (*p* < 0.05) in the obesity group than in the normal and overweight groups (Fig. 2**)**. However, only hypertension prevalence was higher (*p* < 0.05) in WC based abdominal obesity group than in the normal group (Fig. 2**)**.

### Factors associated with obesity and hypertension

Table 3, Table 4 and Table 5 show the risk factors associated with general and abdominal obesity and hypertension from the multivariable logistic regression analysis after adjusting for several covariates. In regression analysis, the female sex, increased age, medium socioeconomic status, low physical activity and place of residence (urban area) were independent risk factors for general obesity (Table 4). The risk of general obesity was significantly higher among subjects aged > 30 years groups compared to the subjects aged 18–30 years group (*p* < 0.05 at least for all cases). On the other hand, female sex, increased age and BMI, low physical activity and place of residence (urban area) were the significant risk factors for abdominal obesity (Table 5). The risk of abdominal obesity was significantly higher among subjects aged > 40 years groups compared to the subjects aged 18–30 years and 31–40 years groups (*p* < 0.01 at least for all cases). For hypertension risk factor analysis, we found a significant association for age, high BMI, low physical activity and place of residence (rural area) with increased risk of hypertension among participants (Table 6). The risk of hypertension was significantly higher among subjects aged > 30 years groups compared to the subjects aged 18–30 years group (*p* < 0.05 at least for all cases).

**Table 4:** Evaluation of the factors associated with general obesity by bivariate and multivariable logistic regression analysis

Variables	COR (95% Cl)	*P*-value	AOR (95% Cl)	*P*-value
Gender				
Male	Ref		Ref	
Female	2.44 (1.78–3.34)	0.000	2.61 (1.88–3.63)	0.000
Age groups (years)				
18–30	Ref		Ref	
31–40	2.13 (1.40–3.22)	0.000	2.00 (1.31–3.06)	0.001
41–50	1.89 (1.17–3.04)	0.009	2.00 (1.23–3.26)	0.005
51–60	1.71 (1.01–2.88)	0.046	1.91 (1.11–3.26)	0.019
≥ 60	2.19 (1.22–3.95)	0.009	2.35 (1.28–4.30)	0.006
WC (cm)				
Normal	Ref		Ref	
Obese	10.01 (6.73–14.89)	0.000	8.65 (5.67–13.18)	0.000
Education				
Above secondary	Ref		Ref	
Secondary	1.30 (0.80–2.12)	0.292	1.17 (070–1.96)	0.549
Primary	1.33 (0.84–2.10)	0.220	1.31 (0.81–2.11)	0.273
Illiterate	2.01 (1.29–3.14)	0.002	1.99 (1.24–3.19)	0.004
Socioeconomic status				
Low	Ref		Ref	
Medium	1.50 (0.97–2.32)	0.066	1.64 (1.04–2.59)	0.032
High	1.48 (0.83–2.63)	0.186	1.54 (0.82–2.88)	0.175
Physical activity				
Adequate/Moderate	Ref		Ref	
Low	1.39 (1.05–2.12)	0.042	1.13 (1.02–1.88)	0.045
Living area				
Rural	Ref		Ref	
Urban	1.73 (1.26–2.38)	0.001	1.92 (1.37–2.68)	0.000
Division				
Barisal	Ref		Ref	
Chittagong	0.52 (0.27–1.01)	0.054	0.46 (0.23–0.92)	0.027
Dhaka	1.41 (0.82–2.44)	0.216	1.32 (0.73–2.40)	0.356
Khulna	0.78 (0.40–1.51)	0.455	0.67 (0.34–1.34)	0.263
Mymensingh	0.72 (0.38–1.39)	0.329	0.57 (0.28–1.17)	0.128
Rajshahi	0.45 (0.23–0.86)	0.016	0.47 (0.24–0.93)	0.031
Rangpur	0.31 (0.14–0.70)	0.005	0.31 (0.13–0.70)	0.005
Sylhet	0.39 (0.19–0.80)	0.010	0.55 (0.26–1.15)	0.112

*COR* Crude odds ratio, *AOR* Adjusted odds ratio, *CI* Confidence Interval

**Table 5:** Evaluation of the factors associated with abdominal obesity by bivariate and multivariable logistic regression analysis

Variables	COR (95% Cl)	*P*-value	AOR (95% Cl)	*P*-value
Gender				
Male	Ref		Ref	
Female	3.14 (2.43–4.06)	0.000	3.51 (2.57–4.80)	0.000
Age groups (years)				
18–30	Ref		Ref	
31–40	1.89 (1.36–2.63)	0.000	1.32 (0.89–1.96)	0.169
41–50	2.16 (1.48–3.14)	0.000	1.91 (1.21–3.01)	0.005
51–60	2.22 (1.47–3.35)	0.000	2.41 91.46–3.96)	0.001
≥ 60	2.56 (1.59–4.14)	0.000	2.80 (1.57–5.01)	0.001
BMI (kg/m^2^)				
Normal	Ref		Ref	
Overweight	5.25 (3.80–7.25)	0.000	5.70 (4.03–8.07)	0.000
Obese	25.14 (16.02–39.44)	0.000	23.00 (14.21–37.23)	0.000
Education				
Above secondary	Ref		Ref	
Secondary	1.30 (0.89–1.91)	0.173	0.91 (0.57–1.44)	0.678
Primary	1.07 (0.75–1.55)	0.699	0.78 (0.50–1.22)	0.277
Illiterate	1.42 (0.97–2.08)	0.075	0.81 (0.50–1.32)	0.401
Socioeconomic status				
Low	Ref		Ref	
Medium	1.25 (0.90–1.74)	0.181	1.34 (0.91–1.99)	0.143
High	0.75 (0.47–1.19)	0.222	0.88 (0.49–1.59)	0.683
Physical activity				
Adequate/Moderate	Ref		Ref	
Low	1.60 (1.17–2.19)	0.004	1.64 (1.11–2.42)	0.014
Living area				
Rural	Ref		Ref	
Urban	1.61 (1.24–2.07)	0.000	1.38 (1.00–1.89)	0.047
Division				
Barisal	Ref		Ref	
Chittagong	0.27 (0.14–0.50)	0.000	0.09 (0.04–0.21)	0.000
Dhaka	1.10 (0.68–1.78)	0.703	0.51 (0.26–1.00)	0.051
Khulna	2.06 (1.17–3.62)	0.012	3.55 (1.67–7.53)	0.001
Mymensingh	1.18 (0.69–2.02)	0.541	2.27 (1.09–4.72)	0.028
Rajshahi	0.55 (0.33–0.92)	0.023	0.87 (0.43–1.75)	0.692
Rangpur	0.69 (0.37–1.28)	0.236	1.33 (0.58–3.04)	0.495
Sylhet	0.74 (0.44–1.26)	0.267	1.70 (0.84–3.43)	0.141

*COR* Crude odds ratio, *AOR* Adjusted odds ratio, *CI* Confidence Interval

**Table 6:** Evaluation of the factors associated with hypertension by bivariate and multivariable logistic regression analysis

Variables	COR (95% Cl)	*P*-value	AOR (95% Cl)	*P*-value
Gender				
Male	Ref		Ref	
Female	1.13 (0.88–1.46)	0.346	1.21 (0.92–1.60)	0.175
Age groups (years)				
18–30	Ref		Ref	
31–40	1.65 (1.13–2.40)	0.009	1.53 (1.04–2.24)	0.029
41–50	3.18 (2.14–4.74)	0.000	2.99 (2.00–4.48)	0.000
51–60	3.99 (2.61–6.10)	0.000	3.75 (2.45–5.76)	0.000
≥ 60	8.18 (4.9–13.61)	0.000	7.91 (4.73–13.23)	0.000
BMI (kg/m^2^)				
Normal	Ref		Ref	
Overweight	1.16 (0.87–1.55)	0.305	1.22 (0.90–1.67)	0.200
Obese	1.73 (1.23–2.45)	0.002	1.57 (1.07–2.31)	0.021
Education				
Illiterate	Ref		Ref	
Primary	0.80 (0.50–1.27)	0.349	0.81 (0.50–1.33)	0.401
Secondary	0.78 (0.48–1.26)	0.311	0.83 (0.50–1.39)	0.478
Above secondary	0.80 (0.54–1.18)	0.254	0.85 (0.56–1.30)	0.454
Socioeconomic status				
Low	Ref		Ref	
Medium	0.84 (0.60–1.17)	0.301	0.87 (0.61–1.25)	0.442
High	1.35 (0.86–2.11)	0.194	1.53 (0.94–2.49)	0.089
Physical activity				
Adequate/Moderate	Ref		Ref	
Low	1.61 (1.13–2.30)	0.008	1.56 (1.03–2.38)	0.036
Family history of hypertension				
No	Ref		Ref	
Yes	0.93 (0.69–1.25)		0.92 (0.67–1.27)	0.625
Knowledge about hypertension				
Yes	Ref		Ref	
No	1.33 (0.99–1.79)	0.060	1.19 (0.85–1.66)	0.304
Living area				
Urban	Ref		Ref	
Rural	1.43 (1.10–1.84)	0.007	1.41 (1.07–1.86)	0.016
Division				
Barisal	Ref		Ref	
Chittagong	1.26 (0.72–2.19)	0.422	1.40 (0.77–2.54)	0.266
Dhaka	0.53 (0.31–0.92)	0.025	0.56 (0.31–1.01)	0.055
Khulna	1.38 (0.77–2.48)	0.282	1.32 (0.71–2.46)	0.380
Mymensingh	1.78 (1.02–3.13)	0.044	1.70 (0.92–3.14)	0.093
Rajshahi	0.99 (0.57–1.71)	0.972	0.92 (0.51–1.67)	0.789
Rangpur	1.64 (0.92–2.91)	0.095	2.27 (1.22–4.23)	0.010
Sylhet	0.68 (0.37–1.22)	0.194	0.81 (0.43–1.53)	0.522
Fried food intake				
Raw salt intake				
No	Ref		Ref	
Yes	0.89 (0.63–1.25)	0.492	0.88 (0.61–1.27)	0.488
Smoking				
No	Ref		Ref	
Yes	0.82 (0.57–1.18)	0.293	0.89 (0.60–1.32)	0.565

*COR* Crude odds ratio, *AOR* Adjusted odds ratio, *CI* Confidence Interval

## Discussion

This study reports on the prevalence of both general and abdominal obesity and hypertension and its associated risk factors in rural and urban residents of all divisional regions of Bangladesh. In this cross-sectional study, the overall prevalence of general obesity, abdominal obesity and hypertension was 18.2, 41.9 and 30.9%, respectively.

In our study, a higher prevalence of general and abdominal obesity was found in women than in men. A similar result was found in studies performed in South India [40] and China [41]. This higher prevalence of obesity in females may be caused by an imbalance of excessive caloric intake and inadequate activity. Moreover, increased parity, menopause, use of high oral contraceptive pills, cessation of alcoholic beverages and smoking might also be contributors to the high prevalence of abdominal obesity in women [42, 43].

We found a high prevalence of both types of obesity in urban participants than in rural participants. The urban participants were more office workers and generally do less physical activity and consume healthier and fatty food which may be related to the increased prevalence of obesity among them. A higher rate of obesity prevalence was also found in urban residents in Myanmar [44] and India [45]. In Bangladesh, a limited number of studies have been conducted to estimate the prevalence of general obesity and abdominal obesity in the general population. A previous study conducted in the Dhaka region of Bangladesh also reported a high prevalence of general obesity (26.2%) and abdominal obesity (39.8%) and this rate was higher in women [26]. A few more studies that used a different anthropometric cut-off value, reported a variation in the range of general and abdominal obesity prevalence in the Bangladeshi population [46, 47]. In our survey, the prevalence rate of abdominal obesity was about twofold higher than general obesity which indicates that an important portion of the study subjects was not identified as obese only based on their BMI levels. Therefore, a specific BMI cut-off level for both sexes may not be enough to estimate general obesity. Considering sex, age and ethnic-specific BMI cut-off levels might be more appropriate for defining general obesity. In risk factors analysis, female sex, increased age, place of residence, and less physical activity were significant predictors of both types of obesity among study subjects. In a previous study, the female sex, increased age and less physical activity were found to be the significant risk factors for obesity in the South Asian region [48].

In the present study, we also found a higher prevalence of hypertension in women than men although the difference was not significant. This higher prevalence in women could be a reason that women have less knowledge about hypertension than men as we observed in this study. Moreover, women are likely to have uncontrolled hypertension in older age [49] and experience increased cardiovascular disease outcomes in later life, mostly due to long life expectancy and hormonal changes [50]. Moreover, women are likely to receive less optimal management for controlling high BP compared with men [51]. In the place of residence comparison, the prevalence rate of hypertension was higher in the rural population than in the urban population. This might be a reason that the rural population were less aware or had insufficient knowledge about hypertension as we observed and generally does not get a better diagnosis and treatment facilities to control hypertension than the urban population. Some early studies in Bangladesh also reported hypertension prevalence in the country but a wide variation has been found between the studies. A systematic review and meta-analysis reported the prevalence of hypertension as 13.5% in the Bangladeshi population [52]. Another study in the country, indicated also a higher prevalence of hypertension (26.4%), with a higher percentage in women than in men [23]. In our study, an increased age, high BMI, place of the living region and inadequate physical activity were independent risk factors for hypertension, and similar findings were also found in some early studies in Bangladesh and studies from other developing countries [23, 53–55]. Of these risk factors, age is an unmodifiable risk factor [56], therefore, in the intervention program concentration should be given to other modifiable factors such as reducing body weight, cutting fatty food from the daily diet menu and being used to some regular physical activity [23]. A significant portion of our study subjects (47.2%) had low or no knowledge about hypertension. Therefore, increasing awareness about hypertension and its health effects would also be effective to reduce the rate of hypertension in the Bangladeshi population. Besides the health workers, electronic and social media can also play an important role in increasing the awareness level of hypertension by broadcasting and sharing videos with simple messages such as what is hypertension and its health effects and its prevention strategies like eating a healthy diet, doing regular exercise, avoiding smoking and alcohol, managing stress and regular blood pressure checkup.

In geographical region comparison, we found a wide variation in the prevalence of obesity and hypertension among study subjects. In our study, the highest risk of general obesity, abdominal obesity and hypertension was observed in Dhaka (central part), Khulna (southwestern part), and Rangpur region (northwestern part), respectively, whereas the lowest risk was found in Rangpur, Chittagong (south-eastern part) and Dhaka region, respectively. These geographic differences might be influenced by dietary food habits, malnutrition and possibly awareness about the diseases. This variation in the prevalence and risk factors of obesity and hypertension in different studies may be related to differences in sampling time points, dietary habits, selection of a specific region, and overall socioeconomic and demographic transition in the last few decades [57, 58]. However, area-specific longitudinal studies are necessary to sort out the underlying causes of the risk of obesity and hypertension across the regions of Bangladesh.

In our survey, we found a higher prevalence of hypertension in both BMI and WC-based obesity groups compared to the normal group. Studies suggested that obesity increases the risk of hypertension and they are occurred often together [15]. We found a significant portion of the participants as obese which may contribute to the increased risk of hypertension and cardiovascular mortality. In addition to obesity and hypertension estimation, we also observed a high prevalence of overweight (38.9%) and prehypertension (40.7%) among study subjects. Both overweight and prehypertension have been recognized as important public health problems. Therefore, regular checking of body weight and blood pressure, eating a balanced diet and daily physical exercise can reduce the future burden of obesity and hypertension among them.

The main strengths of the study were that in this survey we included both gender, a wide age range, place of residence, and all major geographic regions which may reflect the actual scenario about the prevalence of obesity and hypertension in Bangladeshi adults. Furthermore, we collected information on most of the anthropometric and demographic variables that are related to obesity and hypertension. However, our study had some limitations. First, the study was a cross-sectional design and the individual’s blood pressure and obesity data recorded in the survey were from single-day measurement, therefore, a longitudinal study would be useful to identify all possible risk factors of obesity and hypertension for the Bangladeshi population. Second, we had no information on some other important factors like details food habits and lipid levels which may also influence the obesity and hypertension development in the Bangladeshi population.

## Conclusion

Our study showed a high prevalence of general and abdominal obesity and hypertension among Bangladeshi adults. We found that about 1 in 5 adults was general obese, 2 in 5 adults was abdominally obese and about 3 in 10 adults was hypertensive. We observed a wide range of factors which were significantly associated with obesity and hypertension. This study findings indicated that subjects with older age, place of residence and less physical activity were significant determinants of general and abdominal obesity and hypertension. Individuals with lower education and medium socioeconomic status had a higher risk of having general obesity and individuals with high BMI had a higher risk of hypertension. Our findings suggest implying comprehensive and integrated intervention programs to increase awareness and knowledge of obesity and hypertension among both healthcare professionals and patients. These interventions would be modifications of daily lifestyles and food habits at the community level to reduce the burden of obesity and hypertension.

## Data Availability

The datasets used and analyzed during the present study are available from the corresponding author upon reasonable request.
